# The Chromatin Remodelling Factor dATRX Is Involved in Heterochromatin Formation

**DOI:** 10.1371/journal.pone.0002099

**Published:** 2008-05-07

**Authors:** Andrew R. Bassett, Sarah E. Cooper, Anan Ragab, Andrew A. Travers

**Affiliations:** 1 Medical Research Council (MRC) Laboratory of Molecular Biology, Cambridge, United Kingdom; 2 Department of Plant Sciences, University of Cambridge, Cambridge, United Kingdom; 3 Signaling and Functional Genomics, German Cancer Research Center, Heidelberg, Germany; Duke University, United States of America

## Abstract

Despite extensive study of heterochromatin, relatively little is known about the mechanisms by which such a structure forms. We show that the *Drosophila* homologue of the human α-thalassemia and mental retardation X-linked protein (dATRX), is important in the formation or maintenance of heterochromatin through modification of position effect variegation. We further show that there are two isoforms of the dATRX protein, the longer of which interacts directly with heterochromatin protein 1 (dHP-1) through a CxVxL motif both *in vitro* and *in vivo*. These two proteins co-localise at heterochromatin in a manner dependent on this motif. Consistent with this observation, the long isoform of the dATRX protein localises primarily to the heterochromatin at the chromocentre on salivary gland polytene chromosomes, whereas the short isoform binds to many sites along the chromosome arms. We suggest that the establishment of a regular nucleosomal organisation may be common to heterochromatin and transcriptionally repressed chromatin in other locations, and may require the action of ATP dependent chromatin remodelling factors.

## Introduction

Pericentric heterochromatin is a well studied example of a stable condensed chromatin structure that can be inherited from one cell to its daughters, and provides a model system of epigenetic inheritance of a chromatin state. Its occurance correlates with methylation of both DNA at CpG dinucleotides, and histones, particularly tri-methylation of histone H3 on lysine 9 and H4 on lysine 20. One effector of this code is the heterochromatin protein HP-1, which binds preferentially to histone H3 methylated on lysine 9 [1]. We suggest that formation of this condensed state involves the chromatin remodelling factor dATRX and that this protein may act as an effector of the epigenetic code at the pericentric heterochromatin.

Chromatin remodelling enzymes are ATP-driven motor proteins that act to alter chromatin structure and accessibility. They fall into four broad categories – the switch independent / sucrose non-fermenting (SWI/SNF), imitation switch (ISWI), chromodomain (CHD) and INO80-like proteins. The human ATRX protein bears closest homology to the Rad54 class of remodelling enzymes involved in recombination-based DNA repair but has been shown to have chromatin remodelling activity [2] and to be involved in DNA methylation [3] and α-globin gene expression [4]. Indeed, it interacts with the methylated DNA binding protein MeCP2 [5] which is important for its recruitment to methylated DNA.

The human ATRX protein has been linked to heterochromatin function through its direct interaction [6] and colocalisation [7] with the HP-1 protein. It localises to both heterochromatin and promyelocytic leukaemia (PML) bodies in a cell cycle and phosphorylation dependent manner [8]. The homologue of hATRX, *xnp-1*, in *C. elegans* also shows a genetic interaction with the corresponding HP-1 homologue. Recent studies show that PML bodies contain the HP-1 protein, and implicate them in heterochromatin formation in the juxtacentromeric satellite DNA [9].

Several other chromatin remodelling complexes have been implicated in heterochromatin function. A complex containing human Mi-2 has been shown to be recruited to heterochromatin by the Ikaros protein on T-cell activation [10]. Another chromatin remodelling factor (CRF), Brahma related gene 1 (BRG1), has been shown to interact both with HP-1α [11] and the N-terminal region of the human Mi-2 protein [12]. Human ACF has been implicated in replication of heterochromatin during S-phase, and RNAi-mediated depletion causes a delay in cell cycle progression through late S-phase [13]. ACF has also been shown to bind directly to the *Drosophila* HP-1 variant dHP-1a, and aid its loading to chromatin [14]. A recent report shows a physical interaction of the NuRF complex with the heterochromatin protein dHP-2, although the NURF301 subunit failed to show any genetic effect on heterochromatin formation [15]. Finally, purification of a complex containing the methyltransferase Clr3 from *Schizosaccharomyces pombe* revealed the presence of the Mit1 protein, whose chromatin remodelling activity was necessary for heterochromatin formation [16]. Taken together, these results imply a more general role for chromatin remodelling during heterochromatin formation.

A highly sensitive and selective screen for proteins involved in heterochromatin formation is the phenomenon of position effect variegation (PEV). This results from insertion of a gene near to a region of heterochromatin and causes inactivation of the gene in some cells, and activation in others due to variability in the extent of heterochromatin “spreading”. Screens for dominant suppressors of the variegated phenotype have resulted in identification of components involved in establishment or maintenance of heterochromatin, including HP-1 [17].

In this work, we identify novel mutations in the dATRX gene, and show that this gene is involved in heterochromatin formation or maintenance *in vivo* through modification of PEV. We further show that the dATRX protein exists in two isoforms, the longer of which interacts strongly with the *Drosophila* HP-1a protein both *in vitro* and *in vivo*. This interaction is mediated by a CxVxL motif, a variant of a PxVxL motif shown previously to be important for the binding to the chromoshadow domain of HP-1 [6]. The long isoform of dATRX specifically localises to heterochromatin with HP-1a and a mutation in the CxVxL motif abolishes this. Analysis of dATRX distribution on polytene chromosomes shows that the long isoform localises to pericentric heterochromatin at the chromocentre, consistent with its binding to HP-1a, whereas the short isoform is localised to numerous sites along the chromosome arms.

## Results

### The dATRX protein is expressed in two isoforms

The dATRX gene encodes a protein of 148 kDa (1308 amino acids). This is about half the length of the human protein (283 kDa, 2493 amino acids), and is homologous over the C-terminal SWI/SNF-like domain (36% identity), but lacks the N-terminal sequences including the PHD domain found in the human protein.

**Figure 1:**
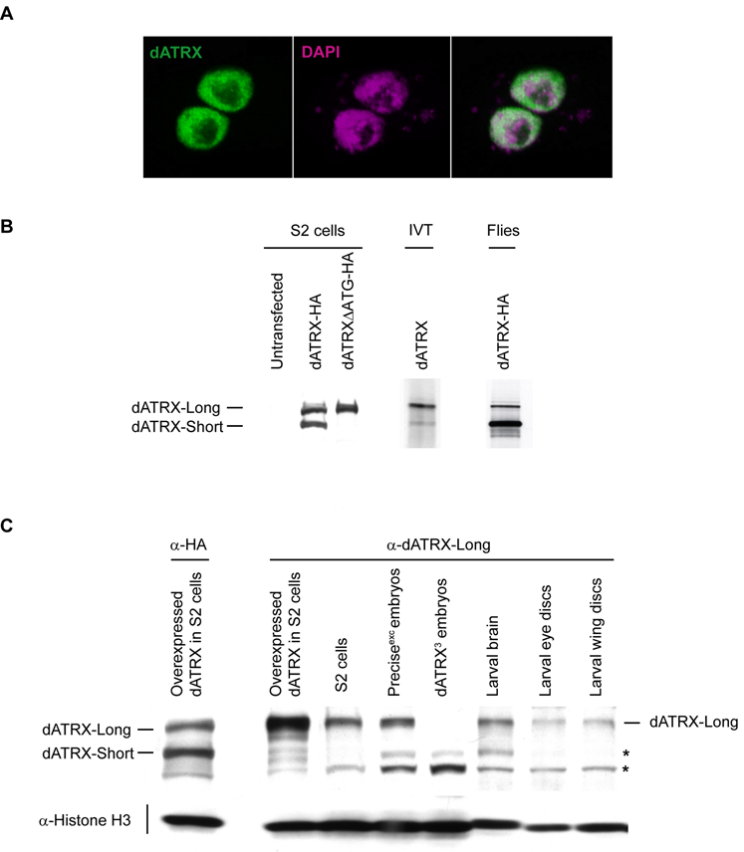
Expression of *Drosophila* dATRX protein. A. dATRX is a nuclear protein excluded from the nucleolus. dATRX with a C-terminal HA tag was transfected into S2 cells, and immunostained (green), and distribution compared to DAPI staining of DNA (purple). B. dATRX is expressed in two isoforms. C-terminally tagged dATRX protein was expressed in S2 cells (S2 cells) or in fly embryos (Flies), and expression analysed by Western blotting. A construct with a mutation in the second start codon at amino acid 266 (dATRXΔATG) lacks expression of the short isoform. dATRX was also expressed by *in vitro* translation (IVT), and showed translation of the two isoforms. C. dATRX-Long is expressed throughout development. When overexpressed in S2 cells, the two isoforms of dATRX were visible on a Western blot probed with anti-HA antibody. When probed with an antibody generated to the first 233 amino acids of the long isoform, only dATRX-Long was detectable in these extracts. Endogenous protein was detected with the same α-dATRX-Long antibody in various tissues. Asterisks (*) indicate non-specific binding. Anti-histone H3 was used as a loading control.

When cDNA was expressed with a C-terminal HA tag in S2 cells, the protein was exclusively nuclear (Figure 1A). Western blotting of such extracts from S2 cells or fly embryos expressing the same construct showed two isoforms (Figure 1B). The same pattern was observed in *in vitro* translation of the cDNA. Mass spectrometry of tryptic peptides identified the shorter form as an N-terminal truncation. There is a second methionine start codon in the protein at amino acid 266, and when this was mutated to an alanine, the short isoform was no longer visible (Figure 1B).

A rabbit polyclonal antibody was raised against the unique N-terminal region of the dATRX-Long isoform. This gave a strong, specific signal corresponding to the long isoform on overexpressed protein in S2 cells (Figure 1C). After affinity purification, endogenous protein was detectable on Western blots of extracts from S2 cells, *Drosophila* embryos and certain larval tissues, but gave significant cross-reactivity when used to detect such low levels of dATRX protein.

### Identification of novel alleles of the dATRX gene

**Figure 2:**
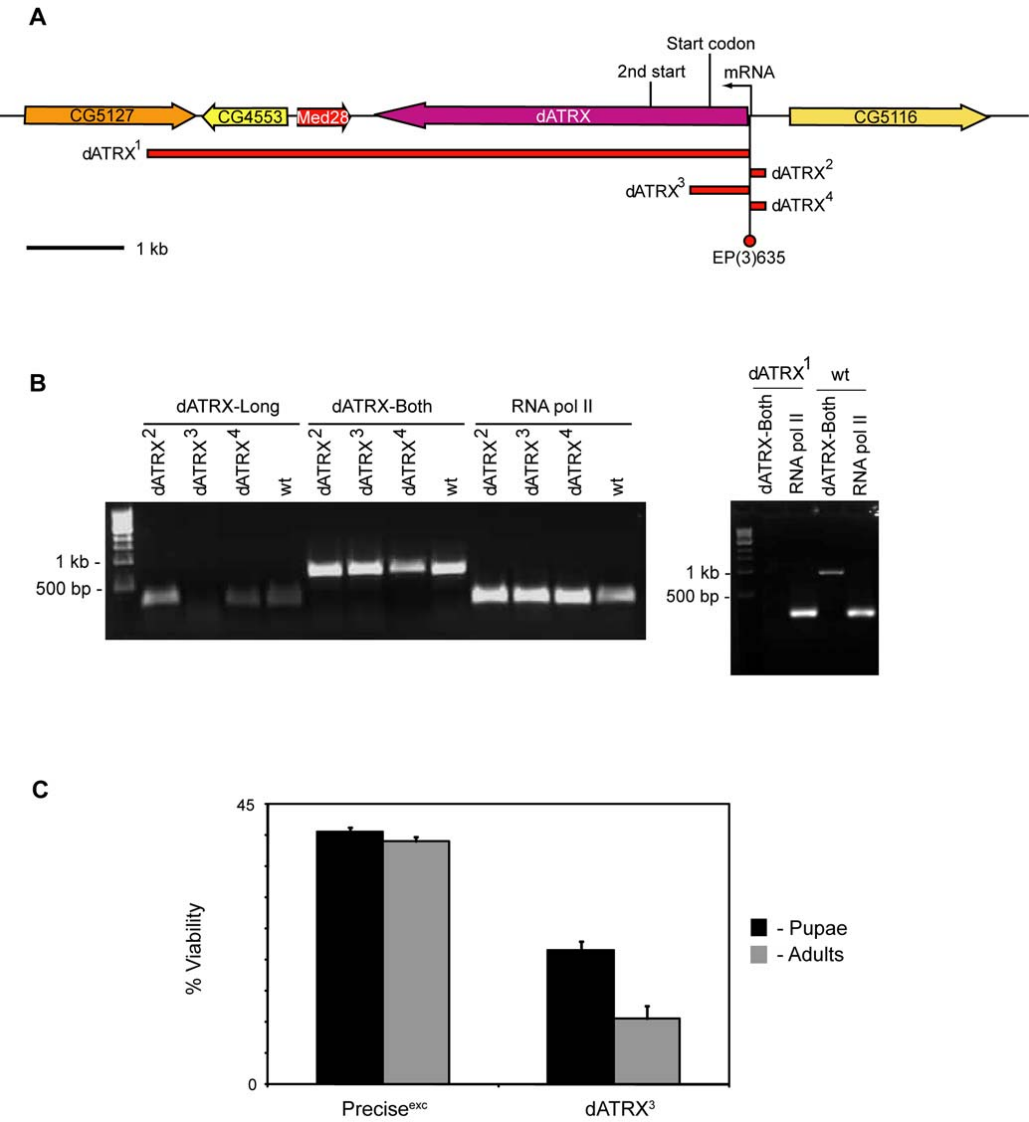
Mutations in the dATRX gene. A. dATRX genomic region showing extent of deletions. The position of the EP element insertion used to generate the deletions is shown (EP(3)635). The extent of the deletions in dATRX^1–4^ are indicated by red bars. Genes are shown as arrows and the start point of the mRNA and protein are indicated (mRNA, start codon). The start codon at amino acid 266 is also marked (2^nd^ start). B. RT-PCR analysis of RNA expression. Homozygous embryos of wild type (wt) or dATRX^1–4^ mutants were analysed for dATRX mRNA expression using oligonucleotides specific for the long isoform (dATRX-Long) or a common region to both isoforms (dATRX-Both). Analysis of RNA polymerase II RNA (RNA pol II) was used as a positive control. Molecular weight markers are indicated. C. dATRX^3^ shows reduced viability. % viability is shown at pupal and adult stages from 100 embryos for both a precise excision (Precise^exc^) and the dATRX^3^ mutation. Error bars indicate standard deviation from two independent experiments.

A EP-element (EP(3)635) present at the beginning of the mRNA, 470 bp from the start of the open reading frame, was used to perform an excision screen for mutants in dATRX. Three semi-lethal lines and one lethal line were isolated. The lethal line, termed dATRX^1^, results from a large deletion of 7.5 kb of DNA including the entire dATRX ORF, the two neighbouring genes, Med28 and CG4553, and some of the 3′ untranslated region of CG5127 (Figure 2A). dATRX^2^ and dATRX4 were semi-lethal and removed 238 bp or 246 bp of the promoter region respectively including the beginning of the dATRX mRNA. dATRX^3^ was also semi-lethal, but removed 786 bp including the start codon of the dATRX gene and the first 105 amino acids of the coding region. RT-PCR analysis of the mutants using primers specific to the long isoform or both isoforms showed that dATRX^2^ and dATRX^4^ still expressed coding sequences corresponding to both isoforms, dATRX^3^ lacked expression of the long isoform, but maintained expression of the short isoform, and dATRX^1^ lacked expression of both (Figure 2B). In embryonic extracts from dATRX^3^ flies, the long isoform was no longer detectable at the protein level on Western blots (Figure 1C). This further verifies the specificity of the antibody. In summary, dATRX^2^ and dATRX^4^ are probably hypomorphic alleles resulting from a promoter deletion, dATRX^3^ removes the long isoform specifically, and dATRX^1^ is a small deficiency covering the dATRX gene and the two neighbouring genes.

Further analysis of dATRX^3^ homozygous flies showed significant reduction in viability at both pupal and adult stages when compared to a precise excision of the P-element (Figure 2C). This suggests that removal of the long isoform causes lethality at many stages during development. Attempts to find specific phenotypes associated with the loss of dATRX-Long have not been successful, perhaps suggesting mild, pleiotropic phenotypes. After several generations, the effect of dATRX^2^ and dATRX^4^ mutations on viability was less evident, so these were not able to be analysed in such a manner.

### dATRX is involved in heterochromatin function

**Figure 3:**
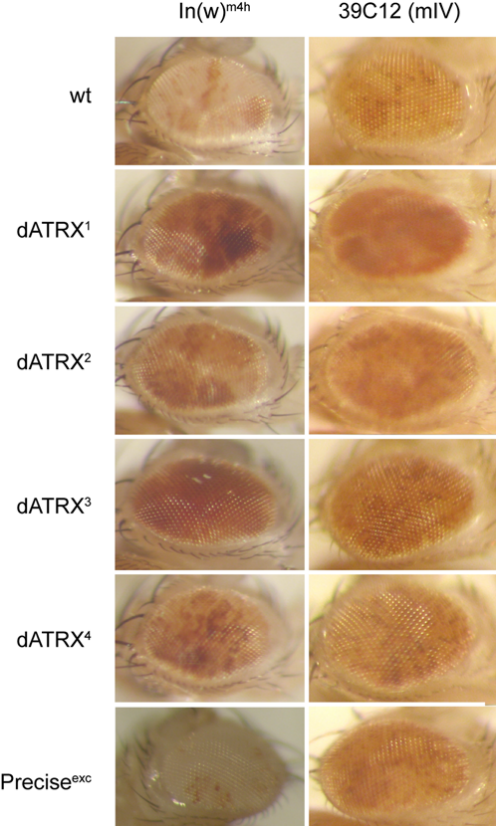
dATRX is involved in heterochromatin formation or maintenance. Flies with the indicated heterozygous mutations were combined with variegating lines on the X chromosome (In(1)w^m4h^) or on chromosome IV (39C12). A cross to wild type flies (wt) and a precise excision of the P-element (Precise^exc^) are shown for comparison of the variegation.

We analysed whether dATRX is involved in heterochromatin formation using an inversion of the first chromosome that places the *white* gene into pericentric heterochromatin (In(1)w^m4h^), which causes variegated expression in the eye. A strong dominant suppression of variegation was observed for all four dATRX alleles (Figure 3) in comparison to both yellow-white flies (wt) and a precise excision of the P-element used to generate the mutants. dATRX^2^ and dATRX^4^ mutations had a weaker effect than the other mutants, consistent with the hypomorphic nature of these alleles. Since the deficiency (dATRX^1^) had a similar effect in this assay to the dATRX^3^ mutation, this may implicate the long isoform in the heterochromatic function of this protein.

A second line showing variegated expression of the white^+^ gene due to insertion into the medial part of the heterochromatic chromosome IV (39C12) [18] was analysed. A dominant suppression of variegation was observed with dATRX^1^ and a mild suppression with dATRX^3^ (39C12, Figure 3), consistent with the above result. No significant effect was seen with dATRX^2^ or dATRX^4^, again suggesting that these are probably hypomorphic alleles.

### dATRX interacts strongly with the heterochromatin protein dHP-1a

**Figure 4:**
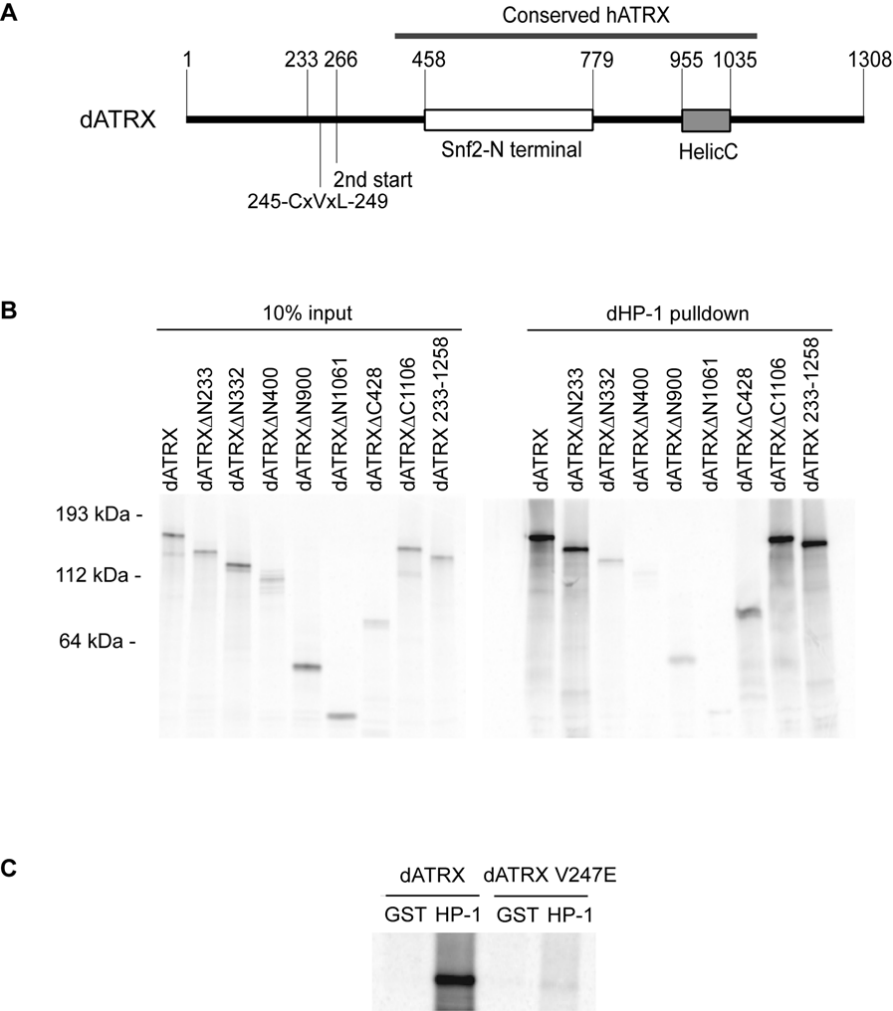
dATRX interacts with dHP-1a directly via a CxVxL motif. A. Domain structure of *Drosophila* dATRX. The Snf2 N-terminal (white box) and C-terminal helicase (HelicC, shaded box) domains are shown. The region interacting with dHP-1a (dHP-1 CxVxL) is also indicated. Numbers show amino acids in the protein. B. Deletion analysis of dATRX binding to dHP-1a. GST pulldowns with GST-dHP-1a and the indicated *in vitro* translated dATRX fragments. Left panel shows expression of the constructs (10% input), and right panel shows GST-dHP-1a pulldown. ΔN and ΔC constructs indicate deletions of amino acids from the N- and C-termini respectively. C. dATRX interacts directly with dHP-1a through a CxVxL motif. Full length *in vitro* translated dATRX proteins containing a wild type CxVxL or a mutant CxExL motif (V247E) were pulled down with GST or GST-HP-1a (HP-1). Strong binding was abolished by the single point mutation.

Given the interaction of human ATRX with heterochromatin protein 1, we asked whether the *Drosophila* protein could also interact biochemically with dHP-1 using a GST-pulldown assay with dHP-1a. Bacterially expressed dHP-1a protein bound strongly to full length *in vitro* translated dATRX (Figure 4B). Analysis of deletion constructs mapped the interaction to amino acids 233–332 (Figure 4B). This region contains a CxVxL motif that is divergent from a consensus PxVxL motif, but which has been shown to mediate interactions between SP100 and the chromoshadow domain of HP-1 [6]. Disruption of this motif in dATRX by mutation of the central valine to a glutamic acid (CxExL) abolished binding to dHP-1a *in vitro* (Figure 4C).

**Figure 5:**
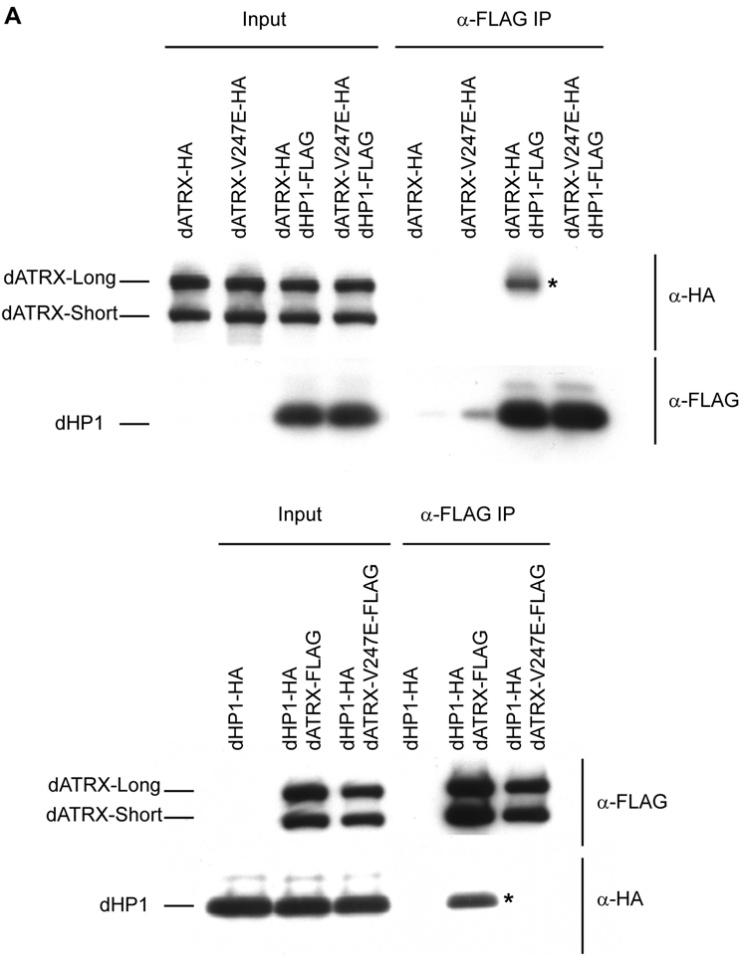
dATRX and dHP-1a interact *in vivo*. A. Co-immunoprecipitation of dATRX and dHP-1a from S2 cells. Cells were transfected with the indicated constructs, and extracts (input) or anti-FLAG immunoprecipitates (α-FLAG IP) were analysed by western blotting for FLAG or HA tags. Co-immunoprecipitating bands are indicated with asterisks.

We then sought to analyse interaction of dATRX with dHP1a *in vivo*. Co-transfection of tagged dATRX and dHP-1a constructs into S2 cells showed a strong interaction between the two proteins when immunoprecipitated with anti-FLAG agarose (Figure 5, asterisks). As expected, this was specific for the long isoform of dATRX, since dHP-1a-FLAG specifically enriched for the long isoform of dATRX when immunoprecipitated from cell extracts (Figure 5, asterisk, upper panel). Mutation of the CxVxL motif in the long isoform of dATRX also abolished the interaction with dHP-1a (Figure 5).

These results are consistent with the ability of the dATRX^3^ allele to strongly suppress PEV, since it removes specifically the long isoform of the protein, which contains the dHP-1a interaction domain.

### dATRX colocalises with dHP-1

**Figure 6:**
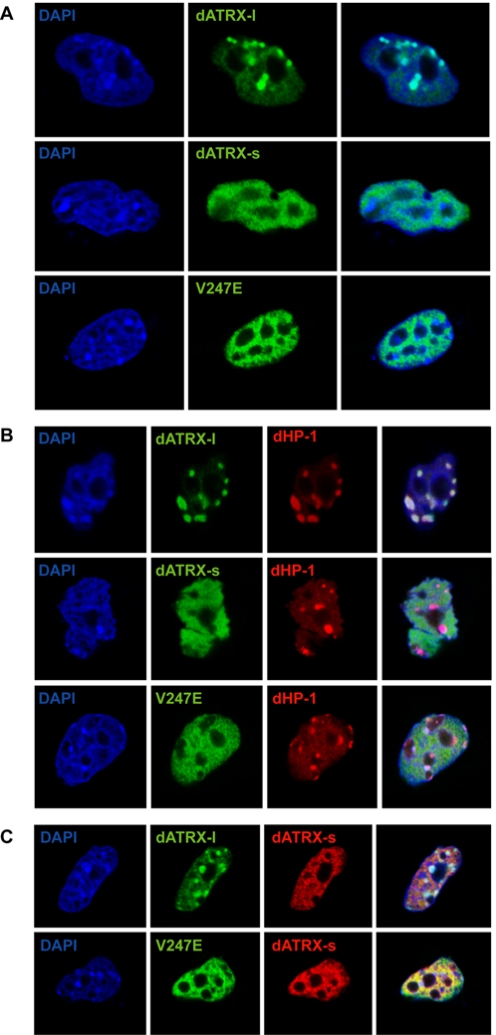
Colocalisation of the long isoform of dATRX and dHP-1a. 3T3 cells were transfected with the indicated constructs, and immunostained with anti-FLAG or anti-HA antibodies. A. Localisation of short (dATRX-s) and long (dATRX-l) isoform of dATRX and a point mutant of the long form removing the CxVxL motif (V247E). DNA staining was performed with DAPI (blue). dATRX localisation was visualised by immunostaining against N-terminal HA tags on the short and long isoforms (green). Right panels show merged images. B. Colocalisation of dATRX with dHP-1a. Cells were co-transfected with HA-dATRX constructs (green) and FLAG-dHP-1a (red), and counterstained with DAPI (blue). Right panels show merged images. C. Localisation of dATRX isoforms. Cells were transfected with the short form of dATRX (dATRX-s) and either the long isoform (dATRX-l) or the CxVxL mutant (V247E, green). Right panels show merged images.

Since dATRX is involved in formation or maintenance of pericentric heterochromatin, and interacts with dHP-1a, we expressed tagged constructs of dATRX and dHP-1a in 3T3 cells to analyse colocalisation of these proteins at heterochromatin during interphase. Expression of the long isoform of dATRX showed localisation to DAPI dense regions of the nucleus (Figure 6A), and upon expression with dHP-1a, showed co-localisation with this protein (Figure 6B). As expected, the short isoform did not localise to the heterochromatin (Figure 6A) or co-localise with dHP-1a (Figure 6B). Indeed, this protein appeared to be excluded from the heterochromatin, and its localisation was non-overlapping with the long isoform (Figure 6C). Upon mutation of the CxVxL motif, the long isoform of dATRX failed to localise to heterochromatin (Figure 6A, B) and showed a similar staining pattern to the short isoform (Figure 6C), showing that the interaction with dHP-1a, or its mouse homologue, is necessary for localisation of the long isoform of dATRX to heterochromatin.

### dATRX staining on polytene chromosomes

**Figure 7:**
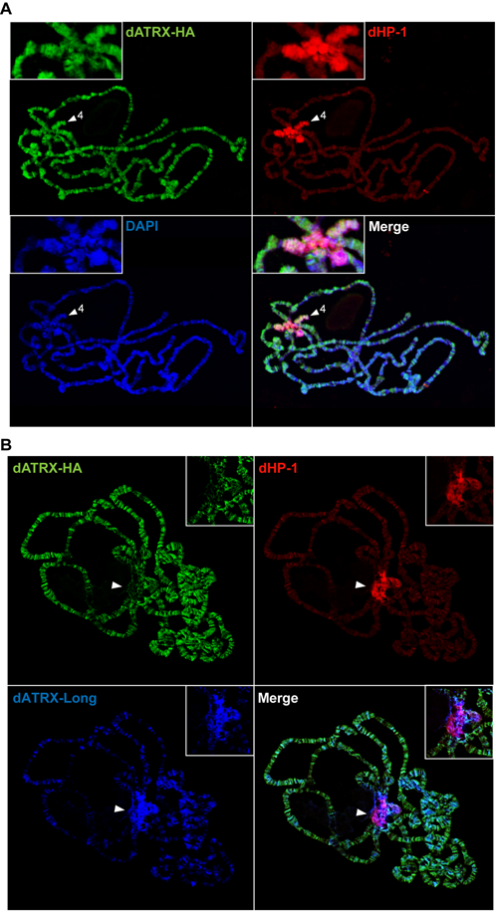
Polytene chromosomes show overlap of dATRX and dHP-1a on chromosome IV and pericentric heterochromatin. A. Polytene chromosome squashes showing double immunostaining for the indicated proteins show overlap between the dATRX-HA protein (green) and dHP-1 staining (red) on the partially heterochromatic chromosome IV (arrowheads). The chromocentre is shown in the enlargements (inset panels). DAPI = DNA staining. B. As (A) but immunostained for the HA tag (green, dATRX-Long and dATRX-Short) and with α-dATRX-Long antibody (blue). Colocalisation of dATRX-Long and dHP-1a is observed at the chromocentre, which is indicated by arrowheads and shown in the enlargements (inset panels).

We analysed distribution of dATRX on salivary gland polytene chromosomes to see whether it was present at the chromocentre, where centromeric heterochromatin is clustered in these nuclei. The protein was overexpressed in salivary glands with a C-terminal HA tag. Detection of both isoforms was performed by immunostaining against this tag (Figure 7A). Staining patterns were compared to those of dHP-1, which stains strongly at the chromocentre. dATRX protein was detected on the partially heterochromatic 4^th^ chromosome (arrowhead, Figure 7A) but predominantly localised to many sites on the chromosome arms. This implies other roles at chromosomal locations other than the pericentric heterochromatin, such as in neuronal specification [19]. Since the protein is overexpressed, we were also able to use the dATRX-Long antibody to specifically detect this isoform (Figure 7B). This showed that the two isoforms differed significantly in their localisation, with an enrichment of the long isoform in the pericentric heterochromatin, colocalising with the HP-1a protein. This is consistent with the binding of dATRX-Long to dHP-1a, and their colocalisation in 3T3 cells.

## Discussion

We have identified novel mutations in the putative chromatin remodelling factor dATRX, and shown that these suppress PEV, using two independent variegating alleles on chromosome I and IV. This implicates the dATRX gene in the process of heterochromatin formation or maintenance *in vivo*.

We further show that the dATRX protein is expressed in two isoforms, the longer of which shows a strong interaction with the dHP1a protein in both GST pulldown and co-immunoprecipitation assays. This interaction is necessary for localisation of the long isoform to heterochromatin in 3T3 cells, and colocalisation with dHP-1a. This interaction is mediated by a CxVxL motif specific to the long isoform, mutation of which abolishes interaction and colocalisation with dHP-1a. Additionally, the long isoform specifically localises to the chromocentre in *Drosophila* polytene chromosomes, providing further evidence for a role of the long isoform in heterochromatin formation. The observed suppression of PEV by the dATRX^3^ allele that removes this isoform specifically suggests that the interaction is relevant *in vivo*. It is also consistent with an observed interaction between human ATRX and HP-1α [6], and genetic interactions between the two homologues in *C. elegans* [20]. These studies combined with the results of the PEV assay strongly imply a role of ATRX in heterochromatin formation in a variety of organisms, and may provide a mechanism of recruitment to such regions.

The exclusion of the short isoform from heterochromatin in 3T3 cells suggests that this has a distinct function at non-heterochromatic sites throughout the genome, and is consistent with its lack of interaction with dHP-1a, and the staining observed in the chromosome arms on polytene chromosomes. Indeed, this staining largely associates with the interbands, representative of less dense chromatin. One role of the short isoform may be during central nervous system formation during embryogenesis, in controlling glial and neuronal patterning [19].

Analysis of the dATRX^3^ mutation that removes the long isoform shows no visible phenotype, aside from reduced viability of the flies. Studies of the chromatin structure in these mutants have failed to show any difference in the nucleosome spacing as judged by micrococcal nuclease digestion (data not shown). This could simply be a consequence of the limitations of this assay or alternatively could suggest a different role in higher order structure formation, or redundancy with other chromatin remodelling factors such as dMi-2.

In order to form a condensed, heterochromatic structure, nucleosome positions must be optimised such that the relative orientations of two nucleosomes are consistent along the fibre. This would allow a regular, ordered structure to form, essential for the formation of a compact fibre and subsequent further folding into a higher order structure such as heterochromatin [21]. *Drosophila* ACF has been shown to act to alter nucleosome repeat lengths both *in vitro* [22] and *in vivo* [23], suggesting a role in “shuffling” of nucleosome positions to generate a more uniform array. One role of the dATRX remodelling factor may be to achieve this. A second mechanism may be by inducing twist, which would aid or antagonise compaction of the chromatin fibre depending on its direction.

We suggest that chromatin remodellers are the end effectors of histone modifications. Consistent with this view, many remodelling complexes contain components that recognise specific modification states of histone tails. For instance, the SANT domain of dISWI may bind to unmodified tails [24], while one of the PHD domains from human Mi-2 binds preferentially to trimethylated lysine 36 of histone H3, which marks the end of active transcription units [25]. ATRX may be recruited by its interaction with HP-1 or MeCP2 [5] to heterochromatin, or the PHD domain in the human protein may play a role in methylated histone binding. In this manner, the epigenetic code present on histones may be translated by chromatin remodelling factors into alterations in folding of the chromatin fibre. Consistent with this idea, the chromatin remodelling activity of Mit1 has been shown to be important for heterochromatin formation in *S. pombe* [16]. We propose that dATRX plays such a role at heterochromatin.

## Materials and Methods

### Immunostaining of S2 cells

Cells were grown on glass coverslips and transfected with FuGENE 6 (Roche). Cells were rinsed in ice cold PBS, fixed in 3.7% formaldehyde in phosphate buffered saline (PBS), for 20 min and washed again. Cells were incubated in 1 ml 0.1% Triton X-100 in PBS for 10 min and non-specific binding blocked by incubation for 2×15 min in PBS+0.5% bovine serum albumin (BSA, Sigma). Primary antibodies were added in the same buffer, and incubated overnight at 4°C. Cells were then washed in 4× PBS+0.5% BSA over 10 min and fluorescently conjugated secondary antibodies were added (Molecular Probes AlexoFluor), and incubated for 2 hours at room temperature. Cells were washed again, stained with 1 µg/ml 4′,6-diamidino-2-phenylindole dihydrochloride (DAPI, Sigma) for 5 min, and mounted in Fluoromount G (Southern Biotechnology). Analysis of staining was performed by confocal microscopy on a BioRad Radiance microscope and images processed using Adobe Photoshop software.

### Polyclonal anti-dATRX-Long antibody production

The N terminal dATRX fragment, 1–233 amino acids (dATRX-N), was cloned into pGEX-4T1 vector (GE Healthcare) to create a GST fusion protein. This was expressed in BL21-DE3 cells for 3 h at 30°C following induction. Protein purification was performed as manufacturer's instructions and the GST tag was cleaved from dATRX-N protein using thrombin. Aliquots of approximately 1 mg/ml dATRX-N protein in PBS were used for injection into rabbits according to standard procedures (Eurogentec). The antibody was affinity purified against GST-dATRX-N protein and used at 1∶1000 for Western Blot and 1∶10 for polytenes.

### Fly stocks and crosses

P-element excision of the EP(3)635 insertion (marked with a w+ gene) was performed by crossing homozygous flies to flies containing the transposase P[Δ2–3], and crossing the resulting EP(3)635/P[Δ2–3] males to w-; TM3/TM6. Stocks were made from the resulting w- males and analysed by genomic PCR across the region around the P-element.

### RT-PCR

mRNA was extracted using Dynabeads mRNA DIRECT kit (Dynal Biotechology) and RT-PCR was performed using the SuperScript III One-Step RT-PCR System (Invitrogen). A PCR reaction lacking reverse transcriptase controlled for DNA contamination.

### Viability test

100 Precise^exc^ and dATRX^3^ embryos (between 0 and 16 h) were allowed to develop at 25°C and the surviving pupae and adults were counted. Two independent experiments were performed.

### PEV

The inversion In(1)w^m4h^ and the white^+^ insertion in the medial region of chromosome IV, 39C12 (a kind gift of S. Elgin) were used to monitor PEV. Yellow^−^ white^−^ (wt) flies and a precise excision of EP(3)635 (Precise^exc^) were used as negative controls. 5–7 day old male flies were used to assess PEV in all experiments.

### GST pulldowns

Radiolabelled proteins were produced using the TnTQuick system (Promega) using plasmid DNA template in the pLinkT7β vector (R. Treisman). Glutathione beads containing 5 µg of the appropriate GST-fusion protein were mixed with 20 µl radiolabelled proteins and 80 µl RIPA buffer, and incubated at 4°C for 1.5 h. The beads were collected by centrifugation at 2000× *g* for 1 min, and washed 4× in 200 µl RIPA buffer. Bound proteins were eluted by addition of 20 µl 3× SDS-PAGE loading buffer and analysed by SDS-PAGE. NuPAGE gels (Invitrogen) were stained and soaked in 30% methanol, 10% acetic acid for 2×5 min before drying. Radiolabelled proteins were analysed by exposure to a phosphorimager cassette (Amersham) overnight, recorded on a Typhoon scanner and manipulated using ImageQuant software (Amersham).

### Co-Immunoprecipiation

S2 cells were transfected with 2.5 µg each of plasmids containing a FLAG-tagged partner and an HA-tagged partner. Expression from the pRmHA3 plasmid (Drosophila Genomic Resource Centre) was induced with 0.7 mM CuSO_4_, and cells incubated for 1 day prior to extract preparation. Cells were collected at 500× *g* for 4 min and washed in 2 ml ice-cold PBS prior to lysis in 200 µl RIPA buffer (100 mM Tris-HCl pH 8, 1 mM EDTA, 0.5 mM EGTA, 1% Triton X-100, 0.1% sodium deoxycholate, 0.1% sodium dodecyl sulphate (SDS), 140 mM NaCl, 1 mM PMSF, Complete protease inhibitors (Roche)) for 15 min at 4°C. Lysates were cleared by centrifugation at 20 000× *g* for 15 min. Immunoprecipitation was performed by addition of 25 µl of a 1∶1 slurry of anti-FLAG agarose (M2, Sigma), and incubation at 4°C for 2 h. Immunoprecipitates were collected by centrifugation at 2000× *g* for 1 min, and washed five times in 0.2 ml RIPA buffer. Proteins were recovered with 1 mg/ml FLAG peptide (Sigma) and samples analysed by Western blotting.

### Immunostaining of NIH 3T3 cells

NIH 3T3 cells were cultured in Dulbecco's modified Eagle's medium (DMEM) (Invitrogen) supplemented with 5% fetal calf serum in a 10% CO_2_ atmosphere at 37°C. Constructs were cloned into pcDNA 3.1 with N-terminal (dATRX long isoform) HA tag or C-terminal (dATRX short isoform) HA or Myc tags or untagged (dHP-1a). For transfection, cells were plated at a density of 1×10^6^ cells per well of a 6 well plate and incubated for 16 h. Cells were transfected with 4 µg DNA, diluted in Optimem (Invitrogen) using lipofectamine 2000 (Invitrogen) according to the manufacturer's instructions. After 5 h the medium was exchanged, and after 2 h recovery, the cells were split onto coverslips. After 16 h cells were fixed and stained as described above for S2 cells. Antibodies used were rat anti-HA (Roche) 1∶500, mouse anti-myc (Sigma) 1∶500 and anti-HP1 (DSHB) 1∶300.

### Polytene staining

Larvae from flies using either heat shock GAL4 (at 25°C) or a salivary gland specific GAL4 line (at 18°C) to drive expression of a UAS-dATRX-HA transgene were used. Salivary glands were dissected in 0.1% Triton X-100 in PBS, then transferred to a drop of 1% Triton X-100, 1.85% formaldehyde in PBS for 10 s. They were then moved to a 40 µl drop of 50% acetic acid, 1.85% formaldehyde in water on a siliconized coverslip for 2 min, and squashed. Slides were washed 2×15 min in PBS and blocked in 10% foetal calf serum, 0.1% Triton X-100 in PBS for 1 h at room temperature. 20 µl of primary antibody solution was added and slides incubated overnight at 4°C. They were washed twice for 15 min in wash buffer 1 (300 mM NaCl, 0.1 Triton X-100 in PBS) and once in wash buffer 2 (1% Triton X-100 in PBS). 20 µl fluorescent secondary antibodies (Molecular Probes) were added for 2 h at room temperature. Washing was repeated, and DNA stained in 1 µg/ml DAPI before mounting in Fluoromount G (Southern Biotechnology) and analysis on a BioRad Radiance confocal microscope. Antibodies used were rat anti-HA (Roche) 1∶50, mouse anti-HP-1a (DSHB) 1∶20 and anti-dATRX-Long 1∶10.
